# MCL-1^Matrix^ maintains neuronal survival by enhancing mitochondrial integrity and bioenergetic capacity under stress conditions

**DOI:** 10.1038/s41419-020-2498-9

**Published:** 2020-05-05

**Authors:** Ujval Anilkumar, Mireille Khacho, Alexanne Cuillerier, Richard Harris, David A. Patten, Maria Bilen, Mohamed Ariff Iqbal, Ding Yuan Guo, Louis-Eric Trudeau, David S. Park, Mary-Ellen Harper, Yan Burelle, Ruth S. Slack

**Affiliations:** 1 University of Ottawa Brain and Mind Research Institute, Department of Cellular and Molecular medicine, University of Ottawa, Ottawa, ON Canada; 2 Department of Biochemistry, Microbiology & Immunology, University of Ottawa, Ottawa, ON Canada; 3 Faculty of Health Sciences, University of Ottawa, Ottawa, ON Canada; 4 Department of Pharmacology & Physiology and Neurosciences, Université de Montréal, Montréal, QC Canada; 5 Department of Clinical Neurosciences, and Cell Biology and Anatomy, Hotchkiss Brain Institute, University of Calgary, Calgary, AB Canada

**Keywords:** Parkinson's disease, Neurodegeneration

## Abstract

Mitochondria play a crucial role in neuronal survival through efficient energy metabolism. In pathological conditions, mitochondrial stress leads to neuronal death, which is regulated by the anti-apoptotic BCL-2 family of proteins. MCL-1 is an anti-apoptotic BCL-2 protein localized to mitochondria either in the outer membrane (OM) or inner membrane (Matrix), which have distinct roles in inhibiting apoptosis and promoting bioenergetics, respectively. While the anti-apoptotic role for Mcl1 is well characterized, the protective function of MCL-1 ^Matrix^ remains poorly understood. Here, we show MCL-1^OM^ and MCL-1^Matrix^ prevent neuronal death through distinct mechanisms. We report that MCL-1^Matrix^ functions to preserve mitochondrial energy transduction and improves respiratory chain capacity by modulating mitochondrial oxygen consumption in response to mitochondrial stress. We show that MCL-1^Matrix^ protects neurons from stress by enhancing respiratory function, and by inhibiting mitochondrial permeability transition pore opening. Taken together, our results provide novel insight into how MCL-1^Matrix^ may confer neuroprotection under stress conditions involving loss of mitochondrial function.

## Introduction

Mitochondria play a central role in cellular homeostasis particularly in cells with high and sustained metabolic rates such as the neurons. In neurons, mitochondria are responsible for a large proportion of total ATP supply, and actively participate in maintaining Ca^2+^ homeostasis, and sustain neurotransmitter release^1–3^. Owing to this central role, mitochondrial dysfunction is implicated in the pathogenesis of a broad range of neurodegenerative disorders^4–6^. In highly vulnerable dopamine neurons, mitochondrially derived oxidative stress is a key contributor to vulnerability and Parkinson’s disease (PD) pathology. Mitochondria are also notably involved in acute neuronal damage induced by oxygen glucose deprivation (OGD)^7,8^ and/or excessive glutamate receptor stimulation^9,10^. These pathological conditions commonly lead to oxidative phosphorylation (OXPHOS) dysfunction, deregulation of Ca^2+^ fluxes, and increased generation of ROS, which damage mitochondrial DNA, proteins, and membrane lipids^11,12^. Damage to these components initiates a vicious cycle of increasing mitochondrial dysfunction that leads to loss of membrane potential, bioenergetics collapse, and activation of apoptotic/necrotic cell death via opening of the permeability transition pore (PTP), a high conductance channel of the inner membrane recently shown to be formed by conformational changes of the ATP synthase^13^. Furthermore, signaling pathways converging on mitochondria under stress can also trigger apoptotic cell death through mitochondrial outer membrane (OM) permeabilization by BCL-2 family pro-apoptotic proteins. Prevention of this vicious cycle constitutes a major therapeutic strategy not only for acute brain injury, but also for degenerative disorders such as Parkinson’s and Alzheimer’s in which mitochondrial dysfunction plays a central role.

MCL-1 is a member of the BCL-2 family anti-apoptotic proteins with intriguing and still poorly understood neuroprotective functions^14–18^. MCL-1 was initially shown to inhibit apoptosis by sequestering the pro-apoptotic BAK^19^, blocking the translocation of BAX to mitochondria^20^ and by interacting with the apoptosis-regulating protein NOXA^21^ on the mitochondrial outer membrane. However, recent studies have identified the existence of two MCL-1 isoforms with distinct intra-mitochondrial localizations, suggesting that this protein may play a broader role than initially predicted. On the outer mitochondrial membrane MCL-1^OM^ antagonizes apoptosis, whereas in the mitochondrial Matrix, MCL-1^Matrix^ has been shown to maintain efficient mitochondrial bioenergetics, optimal assembly of the F1FoATPSynthase oligomers, mitochondrial fusion, and membrane potential^22^. However, whether these distinct mechanisms of MCL-1^OM^ and MCL-1^Matrix^ action play important roles in the regulation of neuronal survival in the context of neurodegeneration and acute injury currently remains unclear.

In this study, we used two models of acute mitochondrial stress to compare and delineate the neuroprotective effects of MCL-1 isoforms. We show that expression of MCL-1^Matrix^ or MCL-1^OM^ in neurons exposed to oxygen/glucose deprivation or glutamate excitotoxicity prevents cell death through distinct mechanisms. Specifically, unlike MCL-1^OM^, MCL-1^Matrix^ preserves mitochondrial OXPHOS and membrane potential. Furthermore, MCL-1^Matrix^ exhibits a remarkable neuroprotective capacity by increasing mitochondrial calcium retention, and by inhibiting mitochondrial permeability pore opening under conditions of neuronal injury.

## Materials and methods

### Neuronal cultures and cell lines

Primary cultures of cortical neurons were prepared from embryonic stage 14–16 CD 1 mice and were cultured for DIV 9–11 as described previously^23^. All experiments were approved by the University of Ottawa’s Animal care ethics committee adhering to the guidelines of the Canadian council on animal care. Human embryonic kidney cells (293T) and MCL-1 ∆/-^16^ cells were grown in Dulbecco’s Modified Eagle’s medium supplemented with 10% fetal bovine serum (Wisnet), penicillin and streptomycin (50 μ/ml), and glutamine (2 mM) (Gibco). Primary cultures of dopamine neurons were prepared from early postnatal (P0-P2) Parkin KO^24^ mice on a confluent astrocyte feeder layer, as previously described^25^. All experiments were approved by the Université de Montréal animal care ethics committee. The seeding density was 100,000 cells/ml. Subsets of cultures were fixed at 1 DIV and others at 11 DIV to examine spontaneous neuronal loss over time in vitro, as previously described^26^. Lentiviral transduction was performed at 1 DIV. Dopamine neurons were labeled using a tyrosine hydroxylase rabbit primary antibody (Millipore, 1:2000) and an Alexa-488-coupled secondary antibody (ThermoFisher).

### Viruses and transfection

Lentivirus carrying GFP, MCL-1^WT^, MCL-1^Matrix^, and MCL-1^OM^^22^ was cloned into WPXLD lentiviral vector and prepared using the AdEasy system. Lentivirus carrying Cherry, Sh scramble Control (5′–CAACAAGATGAAGAGCACCAA-3′)^23^ and mouse specific anti- Sh ATP 5G1 (TRCN0000075774) and ATP 5G3 (TRCN0000305048) were prepared using the ViraPower lentiviral expression system (Invitrogen). For lentiviruses, neurons were transduced with 4 MOI (multiplicity of infection) at the time of plating. MCL-1 ∆/- MEFs were transduced with 8MOI virus with polybreen (8 µg/ml) for 72 h before experimentation. For Cherry, ATP synthase Beta and C-subunits (Origene Technologies) and MCL-1^Matrix^ overexpression experiment, 293T cells were transfected with 5 µg cDNA (unless indicated otherwise) and experiments were performed 72 h post transfection.

### OGD, NMDA toxicity, and cell death measurement

Cortical neurons cultured for 9–11 days in vitro were used to determine neuronal injury following OGD and NMDA excitation. For OGD treatments, culture medium on neurons were exchanged for DMEM medium without glucose supplemented with glutamine, 1% B27 and penicillin-streptomycin and transferred to a hypoxia station (Whitley H35 hypoxystation) with an atmosphere comprising 1% O_2_, 5% CO_2_ and 85% N_2_, with temperature maintained at 37 ˚C. After 90 min of OGD, cultures were returned to oxygenated culture media and allowed to recover for 24 h under normoxic conditions (21% O_2_ and 5% CO_2_).

Cortical neurons cultured for 9–11 days were treated with NMDA/glycine (100 µM/10 µM) for 30 min and washed twice in experimental buffer containing (in mM): 120 NaCl, 3.5 KCl, 0.4 KH_2_PO_4_, 20 HEPES, 5NaHCO_3_, 1.2 Na_2_SO_4_, 1.2 CaCl_2_ and 15 glucose, pH 7.4, supplemented with Mg^2+^ (1.2 mM). Neuronal cell death was assessed 24 h post excitation.

For both OGD and NMDA conditions neurons were stained with Hoechst 33258 at final concentration of 1 µg/µL for 10 min at 37 ˚C. Following the incubation, nuclear morphology was assessed using a Zeiss observer D1 microscope and 20× NA 0.4 objective. Images were taken using AxiocamMRm CCD camera with the appropriate filter sets. For each time point and treatment condensed nuclei were considered as dead and expressed as percentage of total population. Images were processed using FIJI (Wayne Rasband, NIH, Bethesda, MD, USA). Analysis was performed without the knowledge of treatment group.

### Western blot analysis

Western blot were performed as previously described^27^, with following antibodies: mouse anti-actin and anti-flag (Sigma-Aldrich); mouse anti-mtHSP70 (ABR Bioreagents), and rabbit anti-MCL-1 (Rockland). List of antibodies provided in Table S2.

### Mitochondrial isolation

Mitochondrial enriched fraction from neurons were prepared as previously described^28^. The resulting supernatants and pellets (volume equivalents from 10 µg of starting mitochondria) were analyzed by western blot.

### Oxygen consumption

The Seahorse XF24 Extracellular Flux Analyser (Seahorse Biosciences) was used to measure oxygen consumption in cortical neurons. Cortical neurons were seeded onto poly-d-lysine (1 mg/mL) coated 24-well seahorse plates at a density of 1.5 × 10^5^ cells/well in 500 µL neurobasal media supplemented with 1% B27, glutamine and penicillin-streptomycin. Neuorns cultured for 9–11 days were treated with OGD or NMDA as described above. After 24 h post treatment, medium on the neurons were exchanged for HCO_3_-free DMEM medium supplemented with 5 mM glucose, 4 mM glutamine, 1 mM pyruvate and was incubated for 30 min in a CO_2_ free incubator prior to loading into the XF Analyser. Following measurements of basal respiration, neurons were sequentially treated with oligomycin (500 ng/mL) to measure ATP-linked respiration, FCCP (2 µM) to determine maximal respiration capacity and Antimycin A (1 µM) to measure non-mitochondrial OCR. Each measurement was taken over a 2 min interval followed by 2 min mixing and 2 min of incubation. Four measurements were taken for basal OCR and three measurements were taken after oligomycin, FCCP and Antimycin A treatment. All data was compiled by the XF software, normalized to protein levels per well and analyzed with Microsoft Excel.

### ATP luminescence assay

ATP concentration was measured with the CellTiter-Glo® luminescent assay (Promega) using a luminometer (BioTek Synergy H1 hybrid reader) according to manufacturer’s instructions. Data was collected from multiple replicates for each experiment. For each condition ATP concentration was normalized to the number of viable cell as determined by trypan blue exclusion method. ATP-synthase driven ATP was obtained by subtracting oligomycin-insensitive from the total ATP levels.

### Calcium retention capacity assay

MCL-1 ∆/- MEF’s (1 × 10^6^ cells) were resuspended in a surcose buffer (in mM: 250 sucrose, 0.005 EGTA and 10 Tris-MOPS; pH 7.4) containing succinate (5 mM), rotenone (1 µM), and Pi (10 mM). For experiments with CsA (1 µm) and ADP (50 mM), MgCl_2_ (500 µM) plus Oligomycin (100 µM) was added. Changes in extra-mitochondrial calcium concentration was monitored fluorometrically (Hitachi, F4500 spectrofluorometer) using Calcium-green 5 N (1 µM, ex–em: 505–535 nm) as described previously^29^. Residual calcium concentration was adjusted to the similar level at the beginning of every experiment by adding a small amount of EGTA. Calcium pulses, 8.5 µM for MCL-1 ∆/∆ MEF’s were added at 2 min interval until a Ca^2+^- induced Ca^2+^ release was observed. In all experiments, calcium retention capacity was taken as total amount of Ca^2+^ accumulated by mitochondria prior to the Ca^2+^ pulse triggered Ca^2+^ release.

### Confocal microscopy

Primary cortical neurons were loaded with TMRE (20 nM) and Fluo-4 (3 μM) for 30 min at 37 ˚C in the dark in experimental buffer. For Cyclosporin A (CsA) (Sigma-Aldrich) treatment, neurons were pre-treated with CsA (1 μM) for 30 min before the start of imaging. The dish containing neurons were placed on a stage of a Quorum Spinning-disk microscope equipped with Hamamatsu EM CCD digital camera and 60× NA 1.4 objective and a thermostatically regulated chamber maintained at 37 ˚C. On stage cells were treated with 100 µM NMDA/10 µM glycine for 30 min. Images were captured every 60 s. MK-801 (10 µM) was added 30 min after excitation to block NMDA receptor activation. TMRE was excited with 515–560 (BP) and emission was collected at 590 nm (LP). Fluo-4 was excited with 450-490 (BP) and emission was collected at 515 (LP). Corrected total cell fluorescence (CTCF) was quantified using Fiji image analysis software. CTCF was calculated using the formula CTCF = integrated density – (area of selected cell × mean fluorescence of background readings) and was normalized to baseline. Experiments were started with the same amount of baseline acquisition using identical experiment setting between the experiments before the treatment began. Settings were carefully adjusted to avoid under or over exposure during the experiment. For experiments with dopamine neurons, images were captured on a Olympus Fluoview FV1000 confocal microscope and a 60× oil-immersion objective (NA 1.42) on a computer using Fluoview version 3.1b software.

### Immunoprecipitation

Cell lysates were incubated with 1 µg of rabbit anti-MCL-1 (Rockland) antibody in CHAPS containing buffer (50 mM tris HCl, 150 mM NaCl, 1 mM EDTA, 1% CHAPS and 1:1000 PIC at pH 7.4) overnight at 4 ˚C with gentle rocking. Protein A/G beads (Sigma-Aldrich) were added and incubated overnight at 4 ˚C. Following washing (3×) with CHAPS buffer, the bound proteins were eluted with SDS loading dye for 10 min. The resulting blot was probed for MCL-1 and ATP5A with mouse anti-ATP5A (Ab 14748, Abcam). For MCL-1 interaction studies, the human ORF constructs for alpha, beta, gamma, epsilon, and C ATP-synthase subunits, tagged with FLAG and Myc, from Origene Technologies (Rockville, MD) were expressed in 293T cells. Endogenous immunoprecipitation on HEK cells were performed 48 h following transfection. HEK cells were lysed with CHAPS containing buffer and proteins were immunoprecipitated with ANTI-FLAG M2 Affinity Gel (Sigma) according to manufactures protocol. Samples were incubated for 2 h at room temperature and the beads were washed three times with TBS (50 mM tris-HCl, 150 mM NaCl, and 1:1000 PIC at pH 7.4). Immunoprecipitated protein was then eluted from FLAG beads with SDS loading dye for 10 min and analyzed by western blot.

### qRT-PCR

Total RNA was extracted from HEK cells using PureLink RNA Mini Kit (ThermoFisher Scientific) following the manufacturer’s protocol. One-step qRT-PCR gene expression analysis was performed using the rotor-gene SYBR green RT-PCR kit (QIAGEN, 204174). Primer sequences are provided in Table S1. All reactions were run in triplicate or quadruplicate and averaged. Glyceraldehyde 3-phosphate dehydrogenase (GAPDH) was used as internal control for mRNA.

### Statistical analysis

Statistical analysis was performed on GraphPad Prism software (La Jolla, CA, USA). Data are represented as averages ±SD and were analyzed by using one-way ANOVA followed by Tukey’s posthoc test to determine the significance. *P* < 0.05 was considered statistically significant.

## Results

### MCL-1 protects cortical neurons against mitochondrial stress

**Fig. 1:**
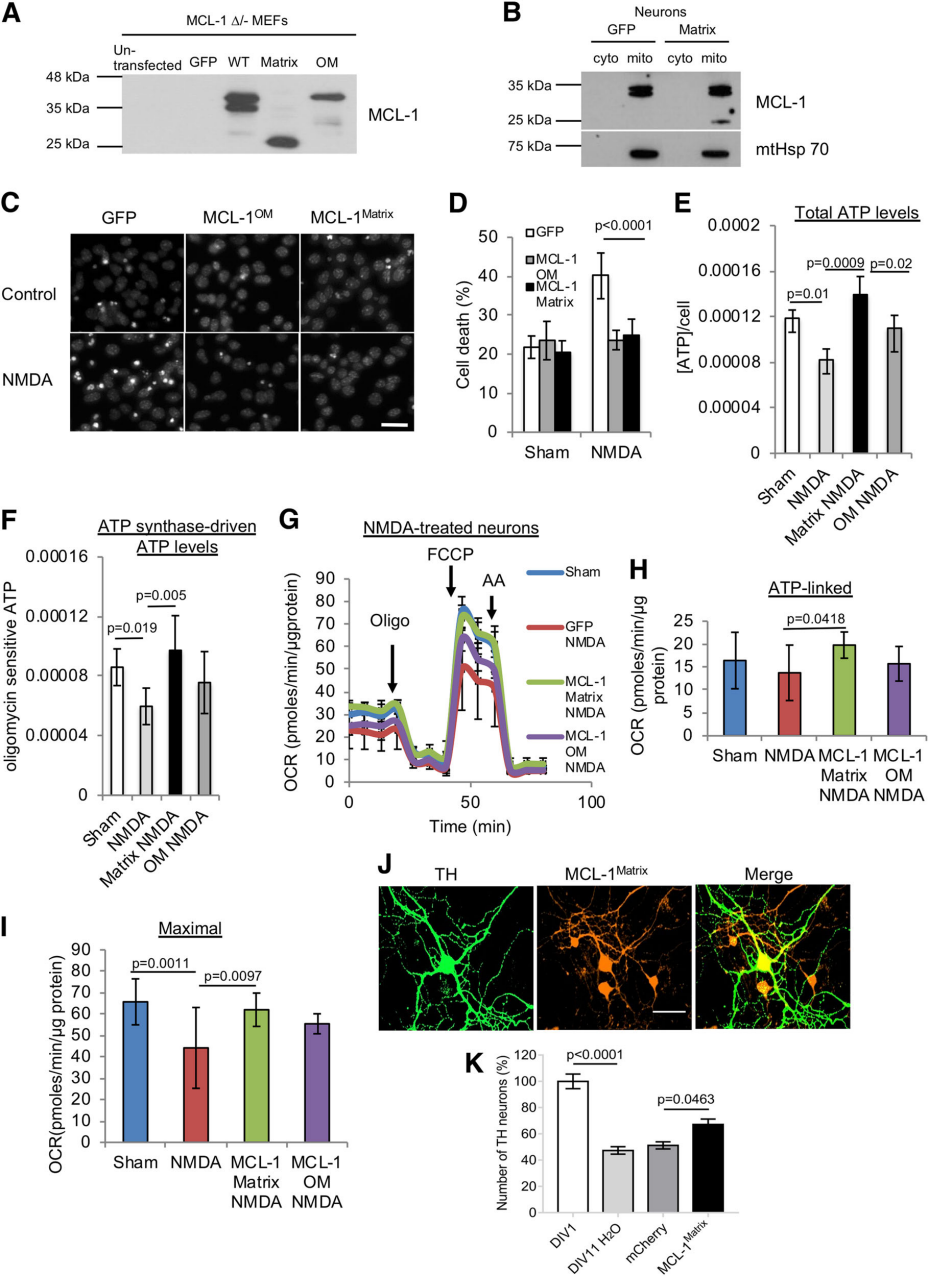
MCL-1 protects neurons against mild mitochondrial stress induced by NMDA excitotoxicity and promotes survival of Parkin KO dopamine neurons. **a** Assessment of MCL-1 expression pattern in MCL-1 ∆/- MEFs transduced with lentivirus carrying GFP, MCL-1 WT, MCL-1^Matrix^, and MCL-1^OM^. **b** Immunoblots from cytosolic fraction (cyto) and mitochondrial-enriched fraction (mito) in WT cortical neurons expressing GFP or MCL-1^Matrix^. Mitochondrial Hsp70 was observed only in mitochondrial fraction. Representative Hoechst images showing healthy (uniformly labeled) and dead (condensed) nuclei in cortical neurons expressing GFP, MCL-1^Matrix^ or MCL-1^OM^ in response to NMDA excitation 24 h post treatment. Scale bar 20 µm. **c** Cortical neurons were transduced as indicated and cell death was quantified in response to NMDA excitation 24 h post treatment (averages ± SD of nine replicates from three independent experiments). **d** Total ATP levels in cortical neurons expressing GFP, MCL-1^Matrix^ or MCL-1^OM^ in response NMDA excitation 24 h post treatment. **e**, **f** ATP in cells subjected to the same conditions as in (**e**) and treated with oligomycin (10 µM) for 1 h. **f** Total ATP levels—oligomycin sensitive ATP levels are shown (averages ± SD of nine replicates from three independent experiments). **g**–**i** Cortical neurons transduced with GFP, MCL-1^Matrix^ or MCL-1^OM^ was treated with NMDA (100 µM/ 10 µM glycine) for 30 min and OCR was measured 24 h post excitation using a Seahorse XF24 Extracellular Flux Analyzer (**g**). Quantification of ATP-linked (baseline OCR minus oligomycin-insensitive OCR) (**h**) and Maximal respiration capacity (FCCP-induced OCR) (**i**) (averages ± SD of nine replicates from three independent experiments). **j** Representative images of primary mouse substantia nigra dopamine neurons from Parkin KO mouse showing tyrosine hydroxylase (TH) in green, MCL-1^Matrix^ co-expressing cherry (red) and overlay of both images. **k** Dopamine neurons were transduced as indicated and survival was quantified at 11 days in vitro (DIV) compared to the number detected at DIV 1 after initial plating to monitor the extent of spontaneous neuronal loss in Parkin KO neurons (averages ± SD from 11–13 coverslips). Scale bar 50 µm. Data information: one-way ANOVA followed by Tukey’s post hoc test.

To investigate the distinct roles of different MCL-1 isoforms, we generated lentivirus-expressing MCL-1^Matrix^ and MCL-1^OM^ as described previously^22^. The MCL-1^Matrix^ construct was generated previously by the Opferman group, fusing N-truncated MCL-1 to the mitochondrial sequence of matrix-localized ATP synthase^22^. This produced a lower molecular weight truncated protein running at ~25 K compared to endogenous MCL-1^Matrix^. Consistent with these results, MCL-1 is expressed as a doublet in WT form (Fig. 1a), mutagenesis to arginine residues at position 5 and 6 of MCL-1 to alanine (MCL-1^OM^) abolishes the generation of relative-molecular-mass (M_r_) 36 K isoform (Fig. 1a) and mitochondrial targeting sequence fused MCL-1 (MCL-1^Matrix^) solely generates the smaller MCL-1 isoform (Fig. 1a) as described previously. The mitochondrial enriched fraction was obtained by differential centrifugation as we have described previously^28^. Western blot analysis of the subcellular fractionation shows mitochondrial markers such that ATP5A, OPA1, and TOM20 are exclusively present in mitochondrial fraction, and AMPK and JNK1 were used as cytosolic markers, and appear in the cytosolic fraction (Fig. S1a). Importantly, we found that MCL-1 is predominantly expressed in the mitochondrial enriched fraction, and MCL-1^Matrix^ resides in mitochondria of neurons expressing this mutant form (Fig. 1b).

Next, to examine the neuroprotective action of MCL-1 isoforms, NMDA-induced excitotoxicity was used in order to induce moderate mitochondrial stress in cortical neurons expressing GFP, MCL-1^Matrix^ or MCL-1^OM^. Neurons were exposed to NMDA for 30 min to induce a robust cellular and mitochondrial Ca^2+^ influx, which is a key trigger of mitochondrial dysfunction and neuronal injury during excitotoxicity^30–32^. After 30 min, Ca^2+^ influx was blocked by high concentration of Mg^2+^ (1.2 mM) containing buffer and neuronal recovery was monitored. As shown in Fig. 1c, d, a twofold increase in cell death was observed in GFP expressing neurons 24 h after exposure to NMDA. In neurons expressing elevated levels of MCL-1^Matrix^ or MCL-1^OM^, cell death induced by NMDA was reduced by 50% (Fig. 1c, d).

The protective effect of MCL-1^OM^ was previously linked to its ability to sequester pro-apoptotic inducers such as BIM and binding to BAX to inhibit mitochondrial OM permeabilization^19,21^. However, the strong neuroprotective effect of MCL-1^Matrix^ was more surprising. Based on its localization within the matrix and its reported role in the maintenance of mitochondrial inner-membrane structure^22^, we reasoned that it might play an important role in maintaining mitochondrial functional integrity.

To test this, mitochondrial function was examined at 24 h post NMDA excitation. We found that in GFP expressing neurons, NMDA caused a 30% reduction in cellular ATP levels and ATP synthase-driven ATP levels (Fig. 1e, f), an impairment of ATP-linked respiration, and a reduction of maximal respiratory capacity (Fig. 1g–i and Fig. S1b–d). While expression of MCL-1^OM^ conferred some protection against bioenergetic dysfunction (Fig. 1d, g, i), the reduction of both basal and maximal respiration was completely rescued in cells expressing MCL-1^Matrix^. Thus, while our results show that both isoforms of MCL-1 are neuro-protective under these conditions, we show that MCL-1^Matrix^ could effectively preserve the integrity of OXPHOS.

Given that MCL-1^Matrix^ was highly efficient at maintaining mitochondrial integrity and OXPHOS in neurons following NMDA treatment, we tested this isoform in another model involving mitochondrial stress, in dopaminergic neurons from Parkin deficient mice. In these animals, mitochondrial function is impaired, in part due to reduced mitophagy, leading to reduced ATP production, increased oxidative stress and mitochondrial fragmentation^33,34^. We reported previously that dopamine neurons from Parkin KO mice show reduced survival in vitro compared to WT mice^26^ and that dopamine neurons are particularly vulnerable in PD because of their very high basal bioenergetic demands and highly active mitochondria due to the extensive size of their axonal arborization^35^. We reasoned that MCL-1^Matrix^ could promote the resilience of nigral dopamine neurons from Parkin KO mice. Confirming this hypothesis, we observed increased survival of Parkin KO dopamine neurons overexpressing MCL-1^Matrix^ but not mCherry control (Fig. 1j, k). Taken together, these results suggest that MCL-1^Matrix^ improves mitochondrial bioenergetic capacity to protect neurons against mitochondrial stress such as Parkin deficiency, or NMDA-mediated excitotoxicity.

### MCL-1^Matrix^ maintains mitochondrial function in acute stress condition

**Fig. 2:**
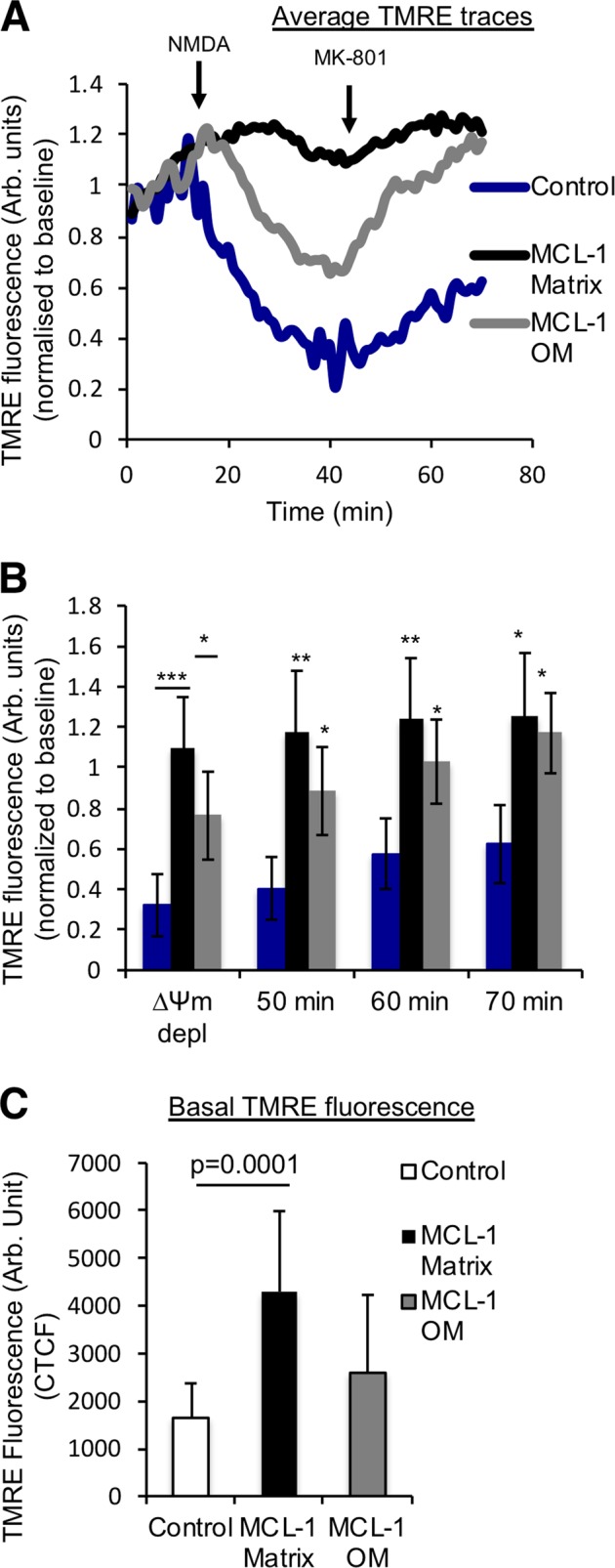
MCL-1^Matrix^ maintains mitochondrial membrane potential in response to acute neuronal stress. **a** Average TMRE traces as in neurons transduced with lentivirus carrying Control, MCL-1^Matrix^ and MCL-1^OM^ during NMDA excitation. **b** Quantification of TMRE traces in (**a**) at the lowest value (∆Ψm depolarization) following NMDA excitation and at different time points as indicated. **c** Quantification of basal TMRE fluorescence in neurons expressing Control, MCL-1^Matrix^, and MCL-1^OM^ (average ± SD of 12–14 neurons from two independent experiments). Data information: one-way ANOVA followed by Tukey’s post hoc test. *P < 0.05, **P < 0.01 and ***P < 0.001.

During excitotoxicity necrotic cell death can occur early due to calcium overload, which causes opening of the PTP, mitochondrial depolarization, and consequent disruption of OXPHOS^36–38^. Exposure of neurons to NMDA leads to overactivation of the NMDA receptor, which disrupts Ca^2+^ homeostasis and immediately depolarized mitochondria (Fig. S2a, b)^39,40^. Application of the NMDA receptor antagonist, MK-801, blocks Ca^2+^ influx into the neurons allowing for recovery of mitochondrial membrane potential. However, the mitochondria that are irreversibly damaged due to excessive Ca^2+^ sequestration cannot fully recovery and undergo mitochondrial permeability pore opening. Consistent with this interpretation, treatment with Cyclosporin A (CsA), an inhibitor of PTP which has been reported to preserve ∆Ψ_m_ following excitotoxic injury^41^, was able to retain mitochondrial ∆Ψ_m_ following exposure to NMDA (Fig. S2c). Alternately, a second window of delayed cell death mediated by pro-apoptotic BCL-2 family proteins can occur several hours after in neurons that were not irreversibly damaged by the initial insult^42,43^. The early response of mitochondrial membrane potential to NMDA was therefore monitored as a mean to distinguish the protective mechanisms of MCL-1 isoforms (Fig. 2a). In GFP expressing cells, ∆Ψ_m_ was reduced by 70% after 30 min of exposure to NMDA, and recovery following blockade of cellular Ca^2+^ entry with MK-801 was partial (Fig. 2a, b) indicating that some organelles had irreversibly depolarized, likely through pore opening. In neurons expressing MCL-1^OM^, a significant decline in ∆Ψ_m_ was also observed 30 min into the NMDA challenge, but the magnitude was smaller, and recovery following blockade with MK801 was complete by 60 min (Fig. 2a, b). In contrast, NMDA-induced depolarization was completely absent in neurons expressing MCL-1^Matrix^ (Fig. 2a, b) indicating that mitochondria effectively took up and retained the Ca^2+^ load without permeability transition. In addition, measurement of ∆Ψ_m_ at baseline also revealed that expression of MCL-1^Matrix^, but not MCL-1^OM^ caused mitochondrial hyperpolarization (Fig. 2c), which favors mitochondrial Ca^2+^ uptake and decreases sensitivity to Ca^2+^-induced PTP opening. Altogether, these results suggested that MCL-1^Matrix^ could protect the bioenergetic integrity of neurons by inhibiting the PTP, either directly, or indirectly by modulating ∆Ψ_m_ or other parameters that affect pore sensitivity to Ca^2+^.

### MCL-1^Matrix^ regulates mitochondrial calcium retention capacity

**Fig. 3:**
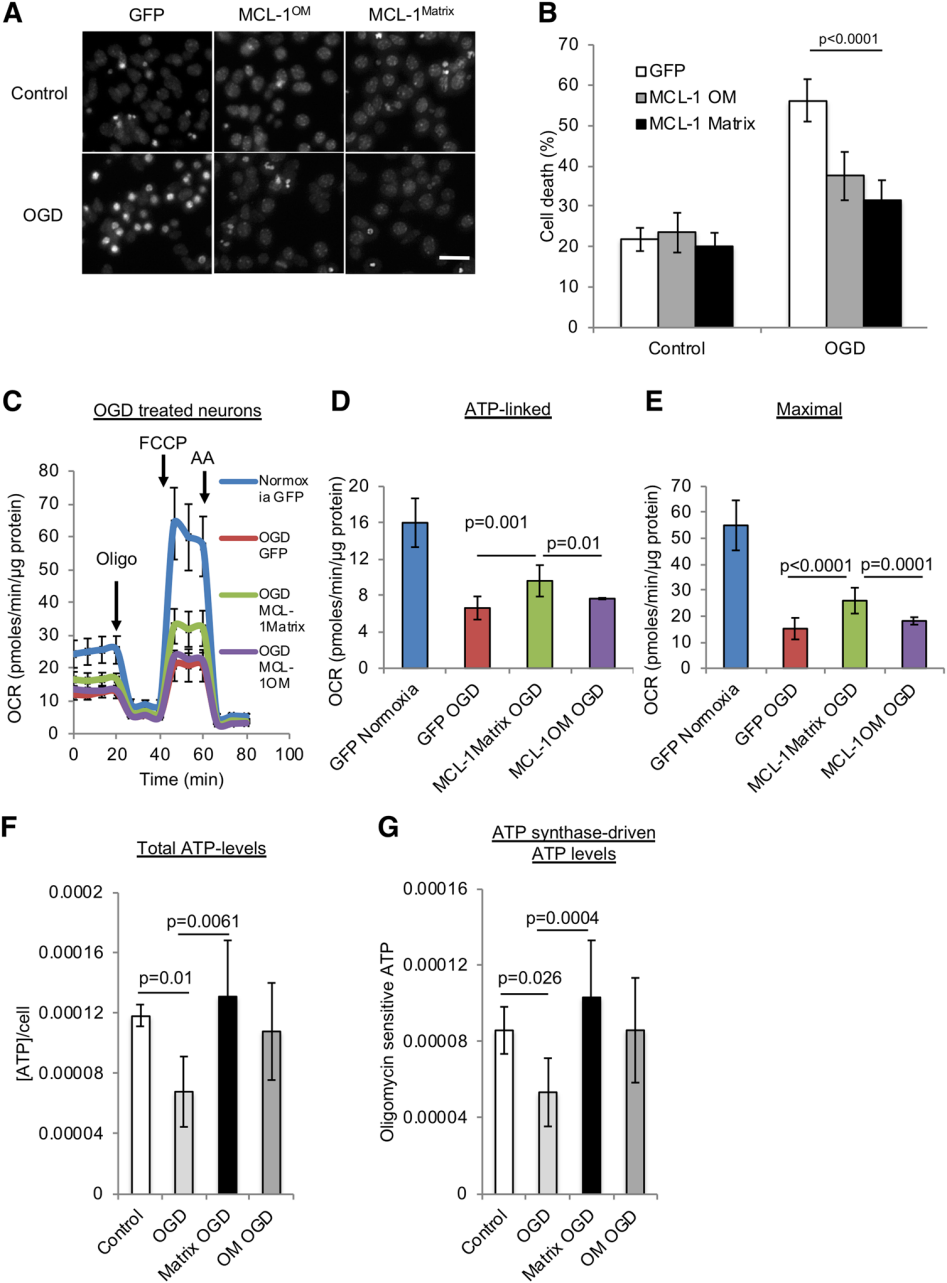
MCL-1^Matrix^ protects neurons and enhances mitochondrial bioenergetics in response to oxygen glucose deprivation. **a**, **b** Representative Hoechst images showing healthy (uniformly labeled) and dead (condensed) nuclei in cortical neurons expressing GFP, MCL-1^Matrix^ or MCL-1^OM^ in response to OGD (**a**) and quantification of cell death is shown in (**b**) (averages ± SD of nine replicates from three independent experiments). **c**–**e** Cortical neurons expressing GFP, MCL-1^Matrix^, and MCL-1^OM^ was treated with either normoxia or oxygen glucose deprivation (OGD) for 2 h and OCR was measured 24 h post injury (**c**). Quantification of ATP-linked (baseline OCR minus oligomycin-insensitive OCR) (**d**) and Maximal respiration capacity (FCCP-induced OCR) (**e**) (averages ± SD of 12 replicates from three independent experiments). **f**, **g** Total ATP levels (**f**) and ATP synthase-driven (Total ATP levels—oligomycin sensitive) ATP levels are shown in (**g**) (averages ± SD of nine replicates from three independent experiment Data information: one-way ANOVA followed by Tukey’s post hoc test.

If MCL-1^Matrix^ acts as a PTP inhibitor, it should also protect from ischemia-reperfusion, a severe stress in which pore opening plays a major role in neuronal cell death^44^. To test this hypothesis, neurons were exposed to oxygen/glucose deprivation for 2 h followed by 24 h re-oxygenation period and cell death was examined. In GFP-expressing neurons, OGD increased cell death threefold (Fig. 3a, b), and severely impaired ATP-linked respiration, maximal respiratory capacity, and cellular ATP levels (Fig. 3c–f and Fig. S3). Cell death was reduced by ~50% in neurons transduced with MCL-1^Matrix^ or MCL-1^OM^ (Fig. 3b). Interestingly, MCL-1^Matrix^ was also able to improve ATP synthase-driven (oligomycin sensitive) ATP levels in response to OGD compared with controls (Fig. 3g). In addition, only MCL-1^Matrix^ was able to limit mitochondrial bioenergetics impairment providing support for a role of MCL-1^Matrix^ in delaying PTP opening.

**Fig. 4:**
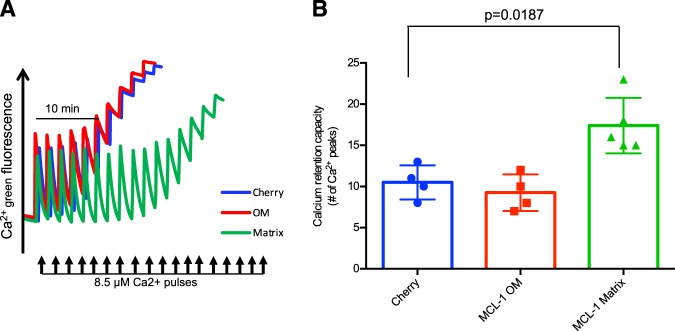
MCL-1^Matrix^ regulates mitochondrial calcium retention capacity. **a**, **b** Calcium retention capacity (CRC) in MCL-1 ∆/- MEFs expressing cherry, MCL-1^OM^ or MCL-1^Matrix^ exposed to consecutive pulses of Ca^2+^ (8.5 µM). Experiments were performed in the presence of Succinate (5 mM), Rotenone (1 µM) and Pi (10 mM) (**a**). Quantification of number of calcium pulses before pore opening (**b**) (average ± SD from four to five independent experiments). Data information: one-way ANOVA followed by Tukey’s post hoc test.

To directly assess whether MCL-1^Matrix^ modulates the PTP, permeabilized MCL-1 ∆/- MEFs expressing MCL-1^OM^ or MCL-1^Matrix^ were exposed to sequential Ca^2+^ pulses every 2 min and the Ca^2+^ threshold for pore opening (i.e., calcium retention capacity (CRC)) was determined. As shown in Fig. 4a, b, CRC was similar in MEFs expressing MCL-1^OM^ or mCherry controls. In contrast, expression of MCL-1^Matrix^ increased CRC by more than 40% compared to mCherry controls and MCL-1^OM^ (Fig. 4a, b), indicating a significant desensitization to permeability transition. Interestingly, CRC values reached in presence of Mg^2+^/ADP and cyclosporin-A were similar in cells overexpressing MCL-1^Matrix^ and mCherry, suggesting that the maximal capacity to delay PTP opening in presence of ATP synthase binding the Fo or lateral stalk is not modified in presence of MCL-1^Matrix^ (Fig. S4).

### MCL-1^Matrix^ regulates mPTP by functional interactions with ATP synthase

**Fig. 5:**
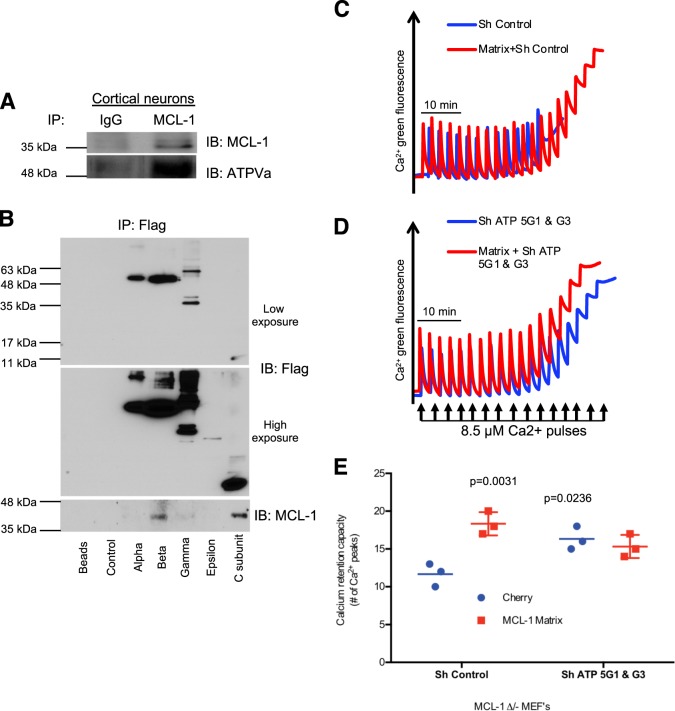
MCL-1^Matrix^ regulates Mitochondrial permeability transition pore through functional interactions with ATP synthase. **a** Endogenous MCL-1 was immunoprecipitated from cortical neurons lysates and the eluted samples were analyzed for MCL-1 and ATP5A expression by western blot. **b** Cherry (control), Alpha, Beta, Gamma, Epsilon, and C subunit of ATP synthase constructs were transiently transfected into 293 T cells. Fourty-eight hours post-transfection, cells were lysed; immunoprecipitated with anti-flag beads and analyzed by western blot. **c–e**) Calcium retention capacity (CRC) in MCL-1 ∆/- MEFs expressing Cherry or MCL-1^Matrix^ and co-transduced with either ShControl or ShATP5G1 & G3. **e** Quantification of CRC shown in (**c**, **d**) (average ± SD from three independent experiments). Data information: one-way ANOVA followed by Tukey’s post hoc test.

As both β and C subunit of the ATP Synthase are believed to be involved in PTP formation we asked if manipulation of the β and C subunit, respectively located on the F1 and Fo subunits, could alter the neuroprotective function of MCL-1^Matrix^. To explore this possibility, we performed immuno-precipitations to seek interactions with the ATP synthase. These experiments showed that ATP synthase co-immunoprecipitated with endogenous MCL-1 in cortical neurons (Fig. 5a). To further examine the potential binding site(s) of MCL-1 on the ATP synthase, recombinant FLAG- and myc-tagged ATP synthase subunits α, β, γ, ε, and C subunit were immuno-precipitated from 293T HEK cells using beads conjugated to anti-FLAG antibodies. These experiments revealed that endogenous MCL-1 co-immunoprecipitated with the β and C subunits (Fig. 5b), respectively located in the F1 and Fo sectors. Both subunits were previously shown to play a role in permeability transition, the β subunit acting as the main Ca^2+^ binding site, and the C subunit as the region involved in pore formation^13,45,46^

To ask whether depletion of C subunits would abolish the ability of MCL-1^Matrix^ to regulate PTP opening, MCL-1 ∆/- MEFs were transduced with lentivirus carrying shRNAs for ATP5G1 and G3 which encode C subunits (Fig. S5). We found that depleting C subunits increased CRC compared with cells transduced with control shRNA, indicating that the presence of C subunits within a normally assembled ATP synthase facilitates pore formation (Fig. 5c–e). MCL-1^Matrix^ overexpression increased CRC in cells expressing control shRNA (Fig. 5d, e). However, in absence of endogenous C subunits, expression of MCL-1^Matrix^ had no effect on CRC, indicating that MCL-1^Matrix^ regulates PTP formation through functional interactions with the ATP synthase, possibly at the level of the C ring.

## Discussion

Regulation of mitochondrial stress-induced neuronal death by BCL-2 family anti- and pro-apoptotic proteins is well established^15,47,48^. However, the distinct roles of the multi-functional, anti-apoptotic protein such as MCL-1 in this context is poorly understood. In this study we provide evidence that compared to MCL-1^OM^, which inhibits canonical pro-apoptotic mechanisms, the matrix isoform preferentially acts to preserve mitochondrial membrane potential and OXPHOS during mitochondrial stress. Furthermore, we show that this effect is linked to the ability of MCL-1^Matrix^ to desensitize mitochondrial PTP formation through functional interactions with the ATP Synthase.

MCL-1 exerts its anti-apoptotic activity by inhibiting pro-apoptotic proteins involved in mitochondrial intrinsic apoptosis pathway^19–21^. Specifically, MCL-1 requires localization to the outer mitochondrial membrane (MCL-1^OM^) for its anti-apoptotic activity^22^. In line with these studies, we show that overexpression of MCL-1^OM^ isoform reduced neuronal death against mild mitochondrial stress conditions. However, MCL-1^OM^ was not effective at preserving OXPHOS integrity. Moreover, during the early response to stress, MCL-1^OM^ isoform was unable to prevent acute depolarization of mitochondrial membrane potential suggesting that it mainly acts during the second window of delayed apoptotic cell death.

In contrast to MCL-1^OM^, MCL-1^Matrix^ has been previously shown to maintain normal mitochondrial inner-membrane architecture/dynamics and regulates mitochondrial bioenergetics^22^. Of note, we have previously demonstrated that restoring mitochondrial architecture either with constitutively expressing mitochondrial fusion proteins OPA1 or Mitofusin 2 protects neurons against mitochondrial stress conditions^49,50^. In this study, we clearly demonstrate that MCL-1^Matrix^ prevents acute depolarization during the early phase of excitotoxic stress and alleviates loss of mitochondrial respiration and ATP production following NMDA and OGD injury. In addition, we also show that MCL-1^Matrix^ protects dopaminergic neurons in Parkin KO, a chronic mitochondrial stress model related to PD. These observations suggest that MCL-1^Matrix^ may protect neurons through a distinct mechanism, by playing a role in the maintenance of mitochondrial function.

An important finding of our study is that MCL-1^Matrix^ desensitizes mitochondria to Ca^2+^-induced PTP opening. Functionally, this results in an improved capacity to maintain mitochondrial membrane potential, OXPHOS and cellular calcium homeostasis during stress. Our results indicate that MCL-1^Matrix^ exerts this modulatory effect on pore opening through functional interaction with ATP synthase. Although the molecular nature of the PTP has been a topic of debate for years. Recent studies have provided strong evidence that the ATP synthase is likely a core structural component^44,51–54^. Various models are currently proposed to explain how the ATP synthase could form the PTP under stress conditions^37,55,56^. The most accepted one is that in the presence of increased Ca^2+^ concentrations and Pi, which are key triggers of pore opening, Ca^2+^ displaces Mg^2+^ from catalytic sites on the β subunits of the F1 sector^44^. Under this condition, the F1 sector undergoes a conformational change that induces pore formation at the level of the transmembrane Fo sector^44^. Although the mechanism remains unclear, this conformational change is believed to propagate from the F1 to the Fo sector via the lateral stalk^13,57,58^ which interacts with several subunits that stabilize the central c-ring in the inner membrane, and assist formation of ATP synthase dimers^57,58^. In this model, binding of cyclophilin-D (the endogenous ligand of the PTP inhibitor Cyclosporin-A), to the OSCP is believed to promote this pore forming conformational change^59^. Our data provides evidence that the C-ring region could be the main site of action of MCL-1^Matrix^. However, at this point, it remains unclear whether MCL-1^Matrix^ binds directly to native C subunit within the fully assembled ATP synthase or whether this binding is indirect, where MCL-1^Matrix^ is associated subunits located nearby. Given the complex nature of PTP formation and regulation, further studies are thus required to establish the interaction of MCL-1^Matrix^ with ATP synthase. A structural analysis of this interaction may provide insights on ways to manipulate PTP opening for the development of neuroprotective mediators in acute brain injury or in diseases such as PD, that are linked to mitochondrial dysfunction.

## Supplementary information

Supplementary Figure and Table Legends

Supplemental Figure S1

Supplemental Figure S2

Supplemental Figure S3

Supplemental Figure S4

Supplemental Figure S5

Table S1

Table S2
