# Supplementary material for: MCL-1Matrix maintains neuronal survival by enhancing mitochondrial integrity and bioenergetic capacity under stress conditions
Source: Cell Death Dis. 2020 May 5;11(5):321. doi: 10.1038/s41419-020-2498-9 (PMC7200794; doi:10.1038/s41419-020-2498-9)
Supplement: Supplementary file 1 — Supplementary Figure and Table Legends [file 41419_2020_2498_MOESM1_ESM.docx]

**Supplementary Figure and Table Legends**

**Figure S1. Subcellular fractionation assessment and Oxygen consumption rate (OCR) in response to NMDA treatment.**

1. Mitochondrial enriched and cytosolic fractions were extracted from neurons and blotted for mitochondrial markers (ATP5A, OPA1 and TOM 20) and cytosolic markers (AMPK and JNK-1) to assess the purity of subcellular fractionation.

(B - D) Cortical neurons transduced LV-GFP were treated with Sham or NMDA and MCL-1^Matrix^ and MCL-1^OM^ transduced neurons were treated with NMDA for 30 min and OCR was determined 24 h post treatment. Quantification of basal OCR (B), reserve OCR (maximal minus baseline) (C) and leak-associated OCR (oligomycin sensitive respiration minus non-mitochondrial respiration) (D) (averages ± SD of 9 replicates from 3 independent experiments).

Data information: one-way ANOVA followed by Tukey’s *post hoc* test

**Figure S2. Mitochondrial membrane potential and Ca^2+^ response in response to NMDA excitation.**

1. Representative images showing TMRE and Fluo-4 staining at t=1 (basal), t=43 (lowest TMRE signal following NMDA excitation) and t=60 (recovery post MK80-1 addition).
2. Average TMRE and Fluo-4 traces in cortical neurons in response to NMDA excitation (average from 14 neurons).
3. Cortical neurons were pre-treated with DMSO or CsA (1μM) for 30 minutes before the start of image acquisition. Average TMRE traces in response to NMDA treatment in DMSO (n = 5) and CsA (n = 4) pre-treated neurons.

**Figure S3. Oxygen consumption rate (OCR) in response to OGD treatment.**

(A - C) Cortical neurons transduced LV-GFP were treated with Sham or NMDA and MCL-1^Matrix^ and MCL-1^OM^ transduced neurons were treated with NMDA for 30 min and OCR was determined 24 h post treatment. Quantification of basal OCR (A), reserve OCR (maximal minus baseline) (B) and leak-associated OCR (oligomycin sensitive respiration minus non-mitochondrial respiration) (C) (average ± SD of 12 replicates from 3 independent experiments).

Data information: one-way ANOVA followed by Tukeys’s *post hoc* test

**Figure S4. MCL-1^Matrix^ regulates mitochondrial calcium retention capacity.**

(A – D) Calcium retention capacity (CRC) in MCL-1 ∆/- MEFs expressing cherry or MCL-1^Matrix^ exposed to consecutive pulses of Ca^2+^ (8.5 µM). Experiments were performed in the presence of Succinate (5 mM), Rotenone (1 µM) and Pi (10 mM) (A) or plus cyclosporine-A (1 µM) (B) or plus combination of ADP (12 µM), MgCl_2_ (0.6 mM) and oligomycin (27 µM) (C). (D) Quantification of CRC in (A - C) (average ± SD from three to four independent experiments).

Data information: one-way ANOVA followed by Tukey’s *post hoc* test

**Figure S5. Validation of knockdown of C subunit of ATP synthase. Related to Figure 5**

293T cells were transduced with Lentivirus carrying ATP synthase C subunit coding genes. Samples were harvested 72 h post transduction. Relative RNA levels of ATP synthase C subunit encoding gene(A) ATP5G1 and (B) ATP5G3 compared to control levels. Average ± SD from three to four technical replicates. All values were normalized to GAPDH levels and expressed as fold change over control condition.

**Table S1. List of primers used in this study.**

**Table S2. List of primary antibodies used in this study.**
