# Supplementary figures and images for: MCL-1Matrix maintains neuronal survival by enhancing mitochondrial integrity and bioenergetic capacity under stress conditions
Source: Cell Death Dis. 2020 May 5;11(5):321. doi: 10.1038/s41419-020-2498-9 (PMC7200794; doi:10.1038/s41419-020-2498-9)

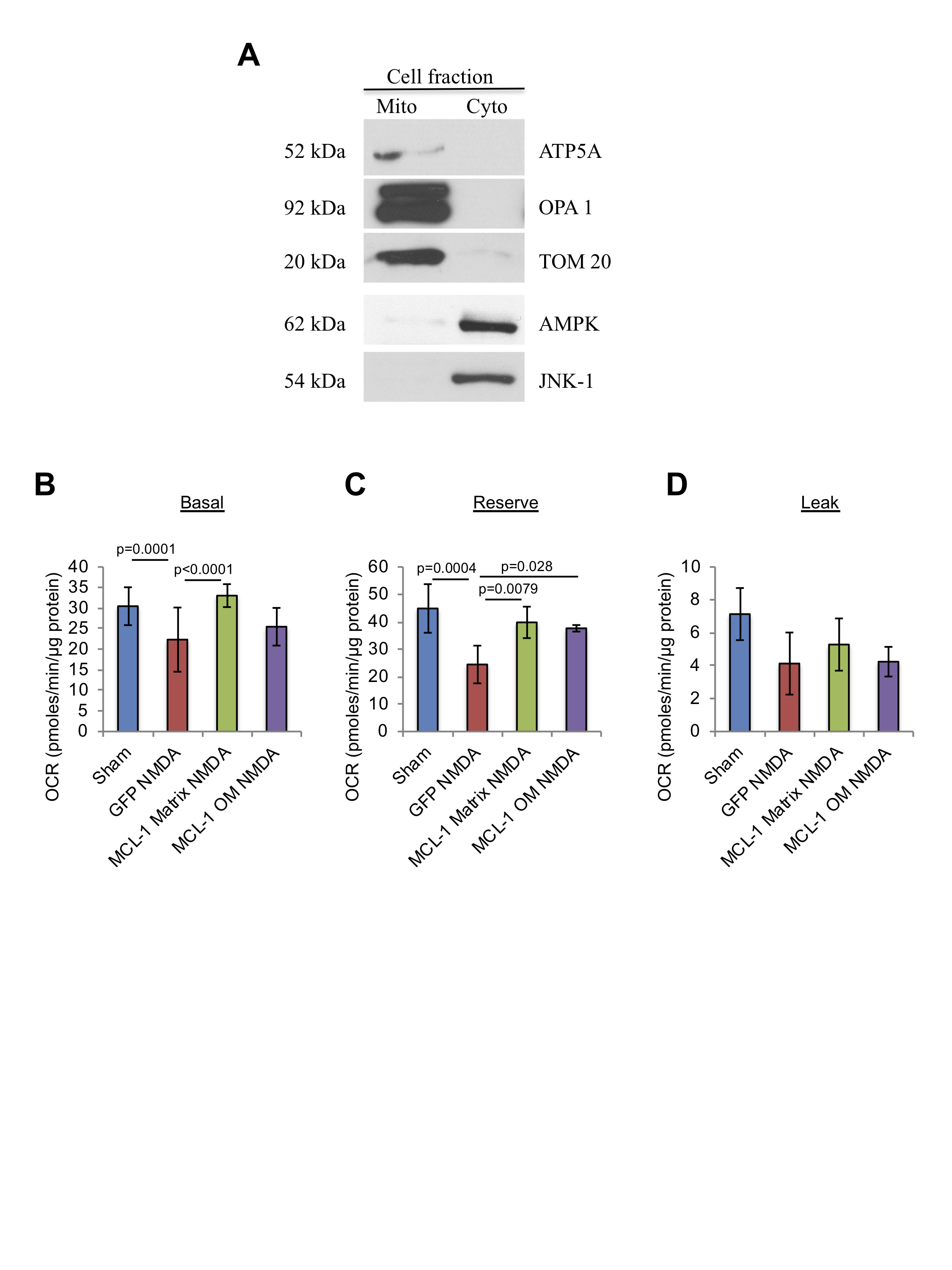

Supplement: Supplementary file 2 — Supplemental Figure S1 [file 41419_2020_2498_MOESM2_ESM.png]

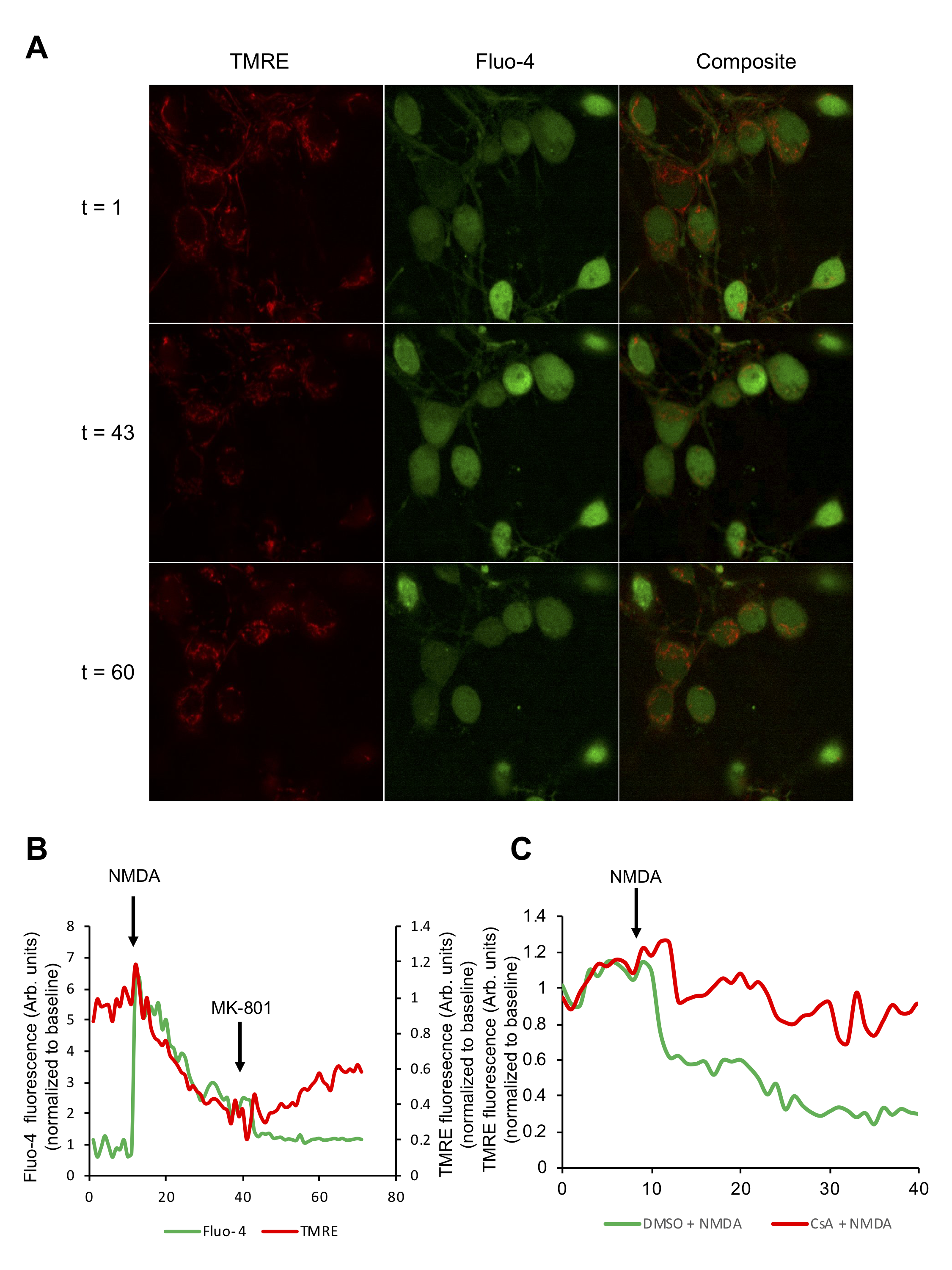

Supplement: Supplementary file 3 — Supplemental Figure S2 [file 41419_2020_2498_MOESM3_ESM.png]

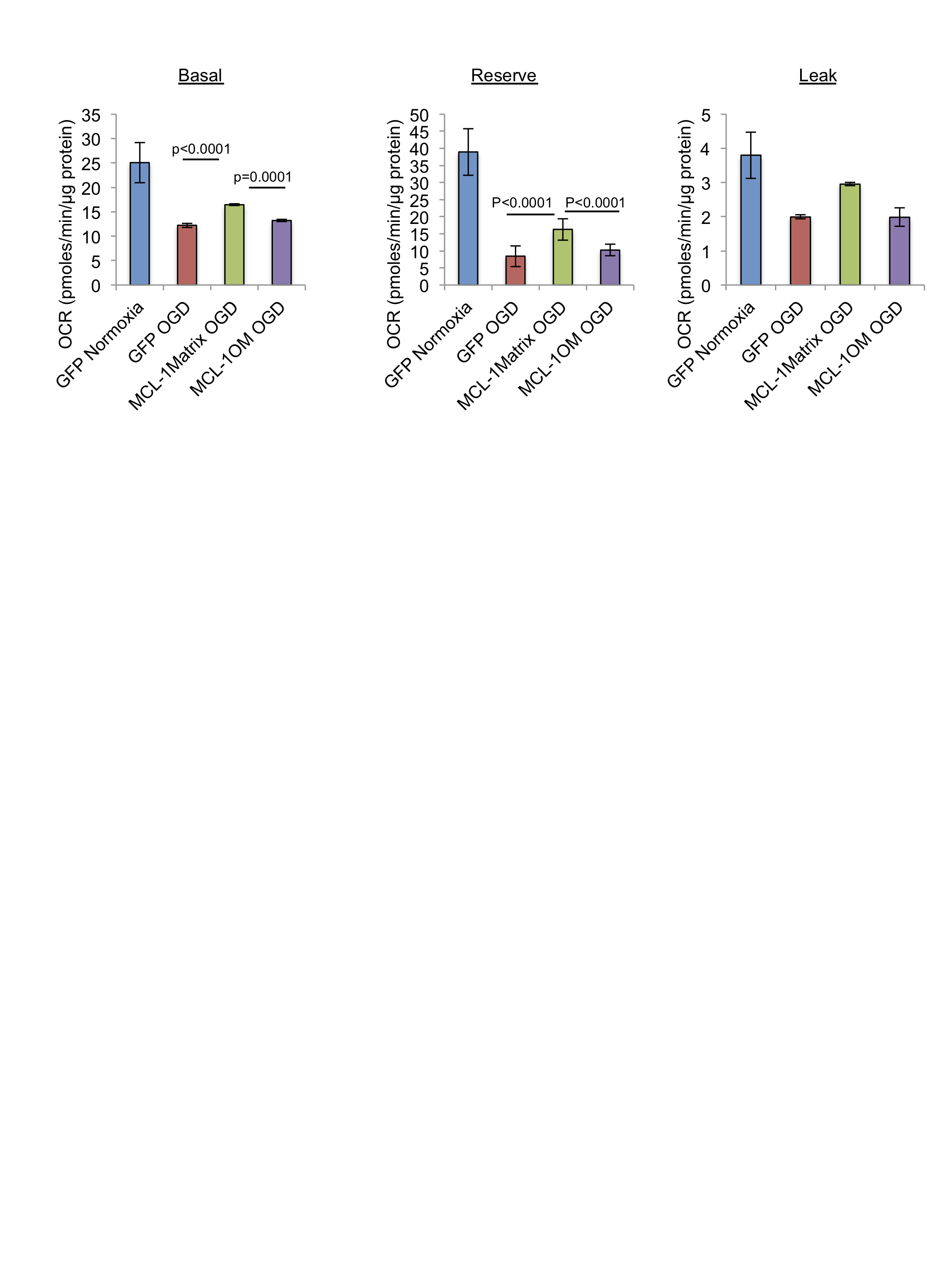

Supplement: Supplementary file 4 — Supplemental Figure S3 [file 41419_2020_2498_MOESM4_ESM.png]

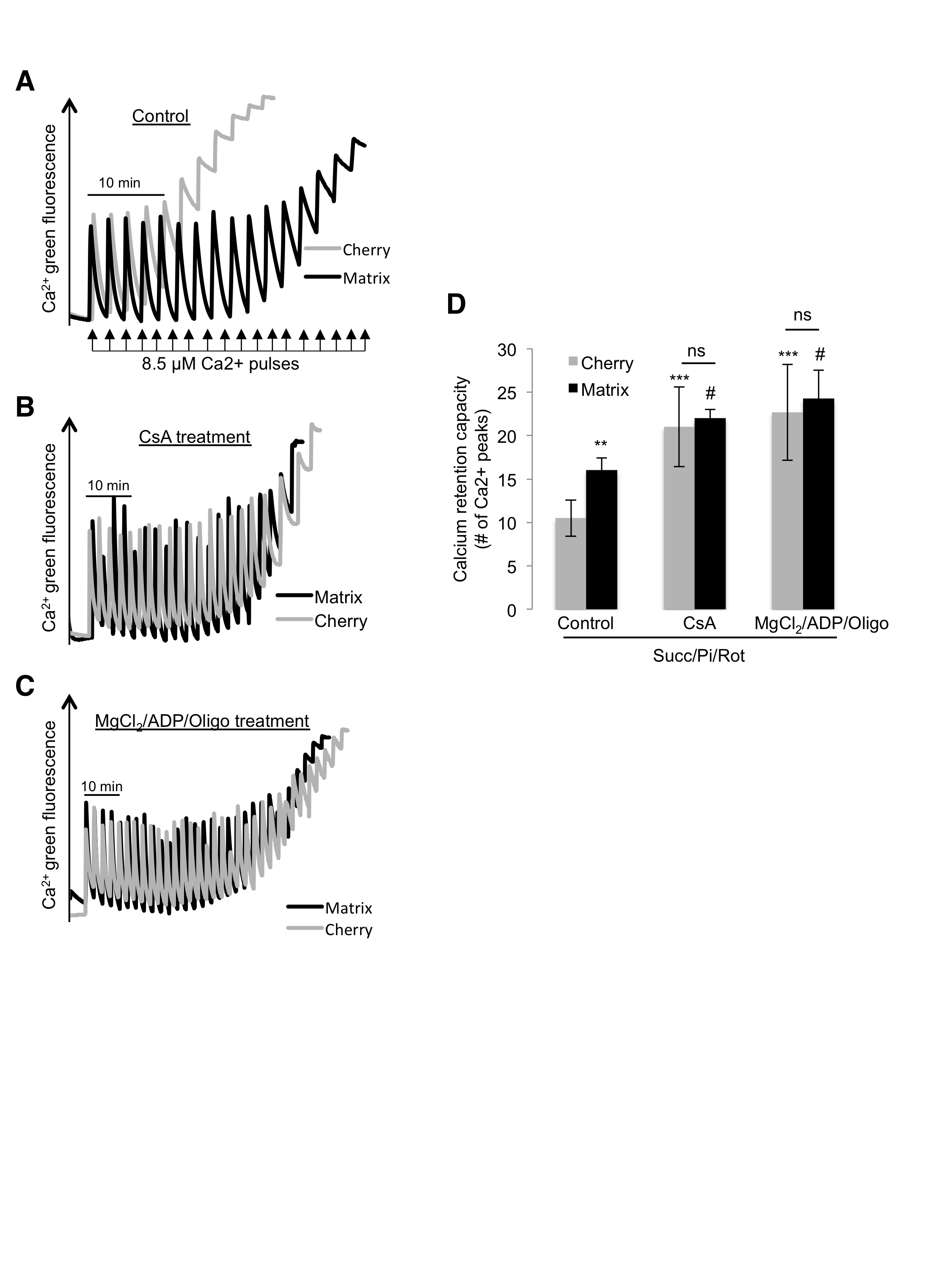

Supplement: Supplementary file 5 — Supplemental Figure S4 [file 41419_2020_2498_MOESM5_ESM.png]

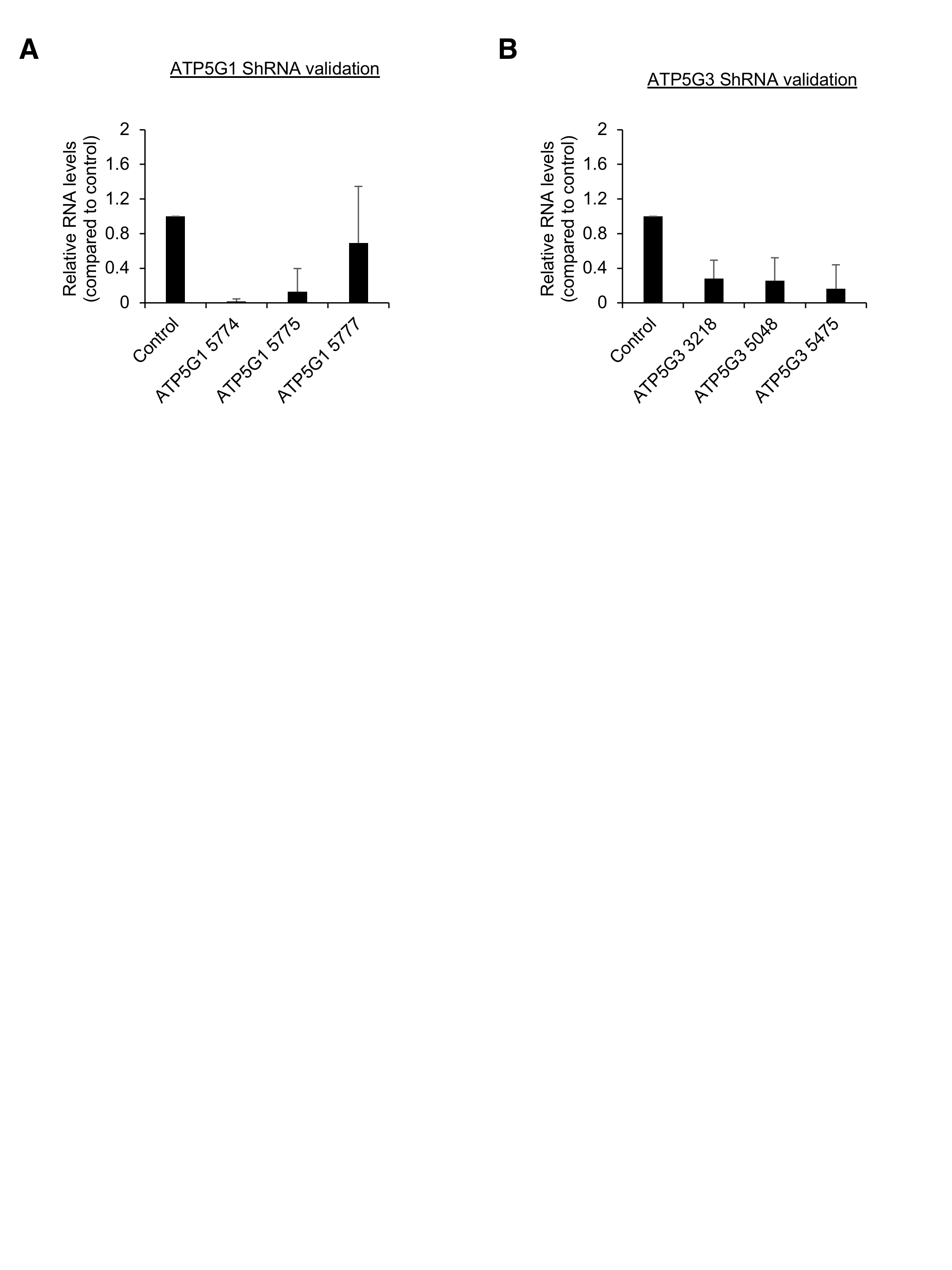

Supplement: Supplementary file 6 — Supplemental Figure S5 [file 41419_2020_2498_MOESM6_ESM.png]
